# Diagnostic yield of medical thoracoscopy: five-year experience at a tertiary care center

**DOI:** 10.36416/1806-3756/e20260200

**Published:** 2026-09-14

**Authors:** Pedro Fernandes, Tito Abrantes, Carla António, Joana Correia, Ana Luís Correia, Beatriz Dias, António Reis, Teresa Belo

**Affiliations:** 1. Centro Hospitalar Tondela-Viseu EPE, Viseu, Portugal.

## TO THE EDITOR:

Medical thoracoscopy (MT) is a minimally invasive procedure that allows direct visualization of the pleural space and biopsy collection, being the gold standard for diagnosing pleural effusions of unknown etiology.^1^ The objective of the present study was to evaluate the diagnostic yield and safety of MT in patients with undiagnosed pleural effusion after initial workup. 

We conducted a retrospective study including all of the patients who, after noninvasive evaluation, underwent MT for pleural effusion of unknown cause at *Centro Hospitalar Tondela-Viseu*, in the city of Viseu, Portugal, between July of 2020 and July of 2025. 

The procedures were performed with patients under deep sedation, which was performed by our anesthesiology teams. The patients were placed in the right/left lateral decubitus, and thoracoscope entry was guided by ultrasound imaging. Biopsies were taken from abnormal areas or random areas (when no abnormalities were present). Talc pleurodesis was performed when indicated, and a chest tube was subsequently placed. 

Clinical, imaging, histological, and follow-up data were collected. Diagnostic yield was primarily based on histological results. 

A total of 32 patients were included (mean age, 68.1 years; male patients, 56%). Most were nonsmokers (75%), and 25% had a history of malignancy. Pleural effusion was right-sided in 59%, left-sided in 28%, and bilateral in 13%. 

The most common endoscopic finding was inflammatory signs (n = 12), with other findings including pleural adhesions (n = 11), nodules (n = 9), infiltrative lesions (n = 4), and pleural mass (n = 1). A mean of 6.25 ± 2.1 biopsies were performed. The mean length of hospital stay was 25 days, and the mean time until chest tube removal was 6.5 days. Talc pleurodesis was performed in 18 patients (56%), and the procedure had to be repeated in 4. 

A histological diagnosis was made in 56.3% of the cases (Table 1). The most common findings were inflammatory infiltrate (43.75%), malignancy (40.63%), and granulomatous disease (15.63%). 

**Table 1:** Histological diagnosis of samples obtained through medical thoracoscopy.

Histopathological findings on biopsy samples	Number of individuals	%
Inflammation • Nonspecific inflammatory infiltrate • Chronic fibrinous pleuritis • Acute necrotizing inflammation	14 7 6 1	43.75%
Malignancy • Lung adenocarcinoma • Mesothelioma • Colorectal adenocarcinoma • Pancreatic adenocarcinoma • Diffuse large B-cell lymphoma • T-cell lymphoma • Breast carcinoma • Prostate adenocarcinoma • Fibrous pleural tumor • Metastatic adenocarcinoma (of unknown origin)	13 3 2 1 1 1 1 1 1 1 1	40.63%
Granulomatous disease • Necrotizing • Non-necrotizing	5 3 2	15.63%

On the basis of clinical history, ancillary test results, and follow-up evaluation, we were able to identify the cause of pleural effusion in 8 of the patients with a histological diagnosis of inflammatory infiltrate. Three patients were ultimately diagnosed with lymphoma on the basis of immunohistochemistry of pleural fluid samples (with 2 being diagnosed by immunohistochemistry of samples obtained through MT and 1 being diagnosed by immunohistochemistry of samples obtained during a previous thoracentesis). One patient was diagnosed with tuberculosis (by means of a positive culture of pleural fluid obtained by thoracoscopy). If we consider those four MT procedures as being diagnostic (although not by means of histology), the overall diagnostic yield of MT was 68.75%. 

Four patients were considered to have pleural effusion secondary to the following: acute cholangitis, in 1-the patient had recently been admitted with acute cholangitis, having undergone endoscopic retrograde cholangiopancreatography and intravenous antibiotic therapy); cirrhosis, in 1-the patient had a medical history of cirrhosis and recurrent right-sided pleural effusion (with no suspicious lesions after extensive ancillary examinations); metapneumovirus, in 1-the patient had recently had pneumonia, having received outpatient antibiotic therapy; and lymphoma, in 1-the patient was undergoing treatment with dasatinib, but the treatment was discontinued after negative findings for malignancy in the diagnostic workup. None of those 4 patients relapsed during follow-up. In the malignancy group, lung adenocarcinoma was the most common diagnosis (n = 3). 

As for the patients with a medical history of malignancy (n = 8), 4 had disease relapse: pancreatic adenocarcinoma, in 1; prostate adenocarcinoma, in 1; breast carcinoma, in 1; and T-cell lymphoma, in 1. 

One patient with adenocarcinoma of unknown origin subsequently underwent upper endoscopy, colonoscopy, and transthoracic biopsies, all of which repeatedly confirmed adenocarcinoma of unknown origin. Despite extensive investigation, the primary tumor site could not be identified. The patient experienced rapid disease progression and ultimately died. 

Granulomatous disease was confirmed to be tuberculosis in 4 patients. After careful investigation and at least two years of follow-up, we were unable to determine the etiology of pleural effusion in 6 patients (5 in the group of patients with a histological diagnosis of inflammatory infiltrate and 1 in the group of patients with a histological diagnosis of granulomatous disease). One patient with acute necrotizing inflammation later had relapsed pleural effusion and was the only patient who underwent surgical thoracoscopy, a diagnosis of chronic fibrinous pleuritis being made. Pleurodesis was then performed, with no relapse during the two-year follow-up period. 

In terms of complications, two cases of infection were identified, resulting in an overall complication rate of 6.25%. Both infections originated at the chest wall, being likely related to the chest tube insertion site. One of the patients progressed to bacteremia, which required prolonged hospitalization and broad-spectrum intravenous antibiotic therapy. Despite being responsive to the antibiotic therapy and experiencing an initial clinical improvement, the patient later died of complications of the underlying disease (pancreatic adenocarcinoma) after 60 days of hospitalization. 

Pleural effusions arise from a wide spectrum of underlying conditions, with congestive heart failure, malignancy, pneumonia, and pulmonary embolism being among the most common causes.^1^ When the initial diagnostic workup fails to establish an etiology, MT represents a cornerstone technique, particularly for exudative effusions.^2^ ^,^ ^3^

The reported diagnostic yield of MT is highly variable, ranging from 40% to 95% depending on the study design, population characteristics, operator experience, and definition of diagnostic success.^4^ ^-^ ^6^ Our overall yield of 68.75% falls within this range but is lower than that reported in larger series. This difference is likely due to our relatively small sample size, and our single-center retrospective design may limit generalizability and increase susceptibility to case-mix variability. Additionally, part of our cohort corresponds to the early phase of MT implementation at our institution, where the operator learning curve and team coordination may have influenced outcomes.^5^ ^,^ ^7^

A high rate of nonspecific inflammatory histology was observed in our study (43.75%), being higher than the typically reported rates.^8^ ^,^ ^9^ From a procedural standpoint, early experience and potential limitations in biopsy targeting or sample size may have contributed to that. Clinically, certain diseases, particularly lymphomas, are known to yield inconclusive pleural biopsies, requiring a combination of cytology, immunohistochemistry, and follow-up data for diagnosis. 

It is of note that nonspecific pleuritis represents a challenge because of its potential association with future malignancy. Studies have suggested that a proportion of such patients may later develop malignancy, particularly mesothelioma. In our cohort, however, no additional malignant diagnoses emerged during a two-year follow-up period, which may reflect adequate initial evaluation or population-specific factors. Nonetheless, our findings reinforce the importance of structured long-term surveillance in such patients.^6^ ^,^ ^9^ ^,^ ^10^

The diagnostic performance of MT was more robust in cases of malignancy and tuberculosis, which are traditionally associated with higher yields because of the presence of visible pleural abnormalities and higher diagnostic sensitivity of histology and microbiology. The inclusion of complementary diagnostic methods increased the overall yield, highlighting the importance of a multimodal diagnostic approach rather than reliance on histology alone. 

Regarding safety, our complication rate of 6.25% is consistent with the literature, which generally reports rates between 2% and 11%. The absence of procedure-related mortality further supports the safety profile of MT when performed in appropriate settings.^3^ ^,^ ^11^ ^,^ ^12^

Overall, our findings confirm that MT is a valuable diagnostic tool, although its effectiveness may be influenced by institutional experience, patient selection, and disease prevalence. Strategies to improve diagnostic yield include more rigorous preprocedural selection using imaging methods (e.g., thoracic ultrasound and contrast-enhanced CT), adoption of advanced biopsy techniques such as cryobiopsy to obtain larger tissue samples, and multidisciplinary case discussion prior to the procedure. 

MT is a valuable and safe diagnostic tool for pleural effusions of unknown origin. Despite a moderate diagnostic yield and a high rate of nonspecific findings, MT remains particularly useful in cases of suspected malignancy and tuberculosis. Improvements in patient selection, technique, and multidisciplinary evaluation may enhance diagnostic performance in the future. 

## References

[1] Light RW (2002). Clinical practice Pleural effusion. N Engl J Med.

[2] Kiani A, Abedini A, Karimi M, Samadi K, Sheikhy K, Farzanegan B (2015). Diagnostic Yield of Medical Thoracoscopy in Undiagnosed Pleural Effusion. Tanaffos.

[3] Gioia M, Arancibia RL (2022). A Review of Medical Thoracoscopy and Its Role in Management of Malignant Pleural Effusion. J Respir.

[4] Dhooria S, Singh N, Aggarwal AN (2014). A randomized trial comparing the diagnostic yield of rigid and semirigid thoracoscopy in undiagnosed pleural effusions. Respir Care.

[5] Khan MA, Ambalavanan S, Thomson D (2012). Comparison of rigid and semirigid thoracoscopes. J Bronchology Interv Pulmonol.

[6] Yu YX, Yang Y, Wu YB, Wang XJ, Xu LL, Wang Z (2021). An update of the long-term outcome of patients with nonspecific pleurisy at medical thoracoscopy. BMC Pulm Med.

[7] Metintas M, Ak G, Dundar E, Yildirim H, Ozkan R, Kurt E (2010). Medical thoracoscopy vs CT scan-guided Abrams pleural needle biopsy for diagnosis of patients with pleural effusions a randomized, controlled trial. Chest.

[8] Davies HE, Nicholson JE, Rahman NM, Wilkinson EM, Davies RJ, Lee YC (2010). Outcome of patients with nonspecific pleuritis/fibrosis on thoracoscopic pleural biopsies. Eur J Cardiothorac Surg.

[9] DePew ZS, Verma A, Wigle D, Mullon JJ, Nichols FC, Maldonado F (2014). Nonspecific pleuritis optimal duration of follow-up. Ann Thorac Surg.

[10] Neilly MDJ, Dick C, Blyth KG (2025). Non-Specific Pleuritis: An Update. Curr Pulmonol Rep.

[11] Deschuyteneer EP, De Keukeleire T (2022). Diagnostic value and safety of thoracoscopic pleural biopsies follow-up of NSP. BMJ Open Respir Res.

[12] Bashour SI, Mankidy BJ, Lazarus DR (2022). Update on the diagnosis and management of malignant pleural effusions. Respir Med.

